# Conventional partial pancreatoduodenectomy versus an uncinate first, extended partial pancreatoduodenectomy approach for the resection of pancreatic head cancer: the randomized, controlled PancER trial

**DOI:** 10.1515/iss-2024-0014

**Published:** 2024-08-26

**Authors:** Patrick Heger, Markus K. Diener, Manuel Feißt, Matthias M. Gaida, Christina Klose, Phillip Knebel, Rosa Klotz, Colette Dörr-Harim, André L. Mihaljevic

**Affiliations:** Department of General, Visceral and Transplantation Surgery, University Hospital Heidelberg, Heidelberg, Germany; Department of General, Visceral and Thoracic Surgery, Nürnberg, Germany; Institute of Medical Biometry (IMBI), University of Heidelberg, Heidelberg, Germany; Institute of Pathology, University of Mainz, Mainz, Germany; The Study Center of the German Society of Surgery (SDGC), University Hospital Heidelberg, Heidelberg, Germany; Department of General, Visceral and Transplantation Surgery, 27203 University Hospital Tübingen, Tübingen, Germany

**Keywords:** pancreatic cancer, pancreatoduodenectomy, pancreatic surgery, extended resection

## Abstract

**Objectives:**

After pancreatoduodenectomy (PD) due to pancreatic cancer, recurrence is frequent in almost half of the patients. The rate of R0 resections is associated with the probability of local recurrence and overall survival. A potential intervention to improve the rate of R0 resections is a more radical resection along the superior mesenteric artery (SMA); however, randomized data of such an approach are lacking. Therefore, we conducted the randomized, controlled PancER trial to evaluate the effect of an extended PD compared with conventional PD.

**Methods:**

Patients were randomized to either an extended PD consisting of a modified Kocher maneuver with partial resection of the prerenal fascia, an uncinate process first approach with systematic mesopancreatic dissection along the SMA equivalent to level III dissection according to Inoue, or conventional PD. The primary endpoint, rate of R0 resections, and other perioperative outcomes were compared.

**Results:**

A total of 50 patients were randomly assigned to extended PD (n=24) or conventional PD (n=26). R0 resections were 10 % more frequent in the extended PD group than in the conventional group (75.0 vs. 64.7 %), which was not statistically significant (p=0.59). Patients self-reported more diarrhea symptoms following extended PD after 30 days (p<0.01). Other perioperative outcomes as well as long-term outcomes were comparable between the two groups.

**Conclusion:**

The PancER trial shows that extended PD with more radical resection along the SMA can be performed with comparable perioperative outcomes to conventional PD. Although the intervention improved the R0 resection rate by 10 %, this increase was below expectation. Therefore, an even more radical PD resection technique involving not only the SMA but also the celiac and hepatic artery (TRIANGLE operation) was developed at Heidelberg University. The TRIANGLE operation is currently being evaluated in a randomized controlled multicenter trial. The results of the PancER trial served as pilot data for this subsequent study.

## Introduction

Pancreatic ductal adenocarcinoma (PDAC) is the fourth most frequent cause of cancer-related death in Germany [1] and the western world, and its incidence is rising [1], 2]. Over 17,000 patients are newly diagnosed with PDAC each year in Germany and almost as many patients die of the disease [1], showing the dismal overall prognosis of pancreatic cancer [1], 2]. Only 15–20 % of patients present with primarily resectable tumors and another 25 % with locally advanced (LAPC) or borderline resectable pancreatic cancer (BRPC) [3]. Furthermore, local recurrence (LR) is frequent after resection of PDAC, and more than 45 % of patients suffer from LR alone or combined with metastases, resulting in decreased overall survival (OS). This trend has been shown in primarily resectable PDAC patients as well as in neoadjuvantly treated patients and/or in BRPC [4], [5], [6]. Microscopic margin clearance (R0) has been identified as an independent risk factor for LR [4], 6]. When using the definition of the R status in PDAC implemented by guidelines [7] including the classification of the circumferential resection margin (CRM) [8], patients with R0 resection show not only lower rates of LR but also better OS rates [4], 9]. Owing to the perineural growth pattern of PDAC, the soft tissue margins toward the celiac artery (CA), superior mesenteric artery (SMA), and the retroperitoneum are the most frequent locations of R1 resections [8], 10]. Thus, most of the LR sites are around the SMA, CA, and the operation bed facing the retroperitoneum, and only 10 % of LR are located in the remnant pancreas [11]. A classification system for the radicalness of resection around the SMA has been proposed by Inoue et al. [12]. More radical resections along the SMA may increase the R0 rate, thereby improving survival. On the other hand, resections of the periarterial nerve plexus of the SMA are associated with severe diarrhea and may impair health-related quality of life [12]. Technically, more radical resections along the SMA can be facilitated by a caudal to cranial resection along the SMV and SMA as proposed by Hackert et al. (“uncinate process first approach”) [13]. The effect of these radical resection techniques along the SMA has not yet been evaluated in a randomized trial.

Therefore, we conducted the randomized, controlled PancER trial to evaluate the effect of extended resection along the SMA compared with conventional PD in patients with pancreatic head cancer.

## Materials and methods

The PancER trial is a randomized, controlled, observer- and patient-blinded, single-center surgical trial with two parallel study groups. The trial was performed at the Department of General, Visceral and Transplantation Surgery of University Hospital Heidelberg in Germany. The trial was performed in line with the IDEAL recommendations [14], and the results are reported according to the current CONSORT guidelines [15]. The trial was started after approval by the local Ethics Committee (project number S-421/2017) of the medical faculty of the University of Heidelberg and conducted in accordance with the Declaration of Helsinki in its current version. The study protocol of the trial was registered at the German Clinical Trial Register (DRKS00013552) and the methods and design of the study were not changed after registration. All surgeons were trained in the PancER intervention before commencement of the trial to minimize the bias raised by the number of surgeons. The results of the trial were initially presented at the 140. German Surgical Congress (DCK 2023) on April, 28th 2023, Munich, Germany.

### Inclusion and exclusion criteria

All patients scheduled for elective PD for suspected PDAC of the pancreatic head with an age of 18 years or more were screened for eligibility. Patients that had undergone neoadjuvant (radio)chemotherapy before surgery were excluded, as neoadjuvant treatment was suspected to significantly influence the R status. Further exclusion criteria were pregnancy or breast-feeding, participation in another interventional trial that might interfere with the intervention and outcomes of this study, and American Society of Anesthesiologists (ASA) higher than grade 3. Only patients with the ability to understand the nature and individual consequences of the clinical trial were included after giving written informed consent.

### Randomization and blinding

In order to achieve comparable groups, randomization was performed using the centralized online randomization system www.randomizer.at from the Medical University of Graz. The online randomization procedure provided information regarding the group allocation and a randomization number. The randomization sequence was computer-generated using the standard continuous uniform distribution. Equal blockwise randomization was performed in the operating room after surgical exploration and assessment that resection of the tumor via PD was possible. Furthermore, intraoperative confirmation was needed that the tumor suited both interventions, leading, among others, to exclusion of cases with arterial involvement.

Patients and outcome assessors were blinded to the intervention in order to guarantee unbiased assessment of the primary and secondary endpoints. Patients were blinded by virtue of being under general anesthesia during the trial intervention. The primary outcome assessor (pathologist) was neither part of the surgical team that performed the trial intervention nor did they have access to the randomization tool. Similarly, secondary endpoints were assessed by blinded outcome assessors who were neither part of the surgical team that performed the trial intervention nor had access to the randomization tool. Health-related quality of life was assessed by the blinded patients themselves via validated questionnaires.

### Intervention of the experimental and control group

The perioperative and intraoperative treatment of the intervention and the control group were the same with the exception of the resection phase during surgery. After exploratory laparotomy and exclusion of hepatic or peritoneal metastases, in both groups dissection of the gastrocolic ligament and definition of the tumor extent in the lesser sac was performed, followed by a standard Kocher maneuver of the duodenum. After exclusion of para-aortal lymph node metastases, the procedure was continued according to the randomization. Lymphadenectomy was performed in both groups in accordance with current guidelines [16].

The experimental intervention consisted of (see Supplementary Material 1.1 and 1.2):

(1) A modified Kocher maneuver as described by Weitz et al. with partial resection of the prerenal fascia [17].
(2) An uncinate process first approach as described by Hackert et al. [13].
(3) A systematic mesopancreatic dissection (SMD) along the SMA from at least 5.00 o’clock to 11.00 o’clock (≥180°), equivalent to level III dissection according to Inoue et al. [12].

Therefore, patients in the experimental group underwent wide mobilization of the duodenum until the anterior surface of the inferior vena cava and the aorta were exposed, followed by and partial resection of the prerenal fascia to enhance posterior margin clearance. During the resection in the intervention group, a caudo-cranial mobilization of the uncinate process (uncinate first) was used, which included central ligation of the inferior pancreatoduodenal artery (IPDA), the first jejunal artery (JA) (and, if necessary, the second JA), the jejunal vein (JV), the inferior pancreatoduodenal vein (IPDV), and Henle’s gastro-colic trunk (HGCT). Around the SMA, dissection of the nervus plexus from at least 5 o’clock to 11 o’clock (180°; right hemicircle of the SMA) as described by Inoue et al. (level III) was performed [12], with additional circular (360°) dissection of the SMV.

In the control group, a standard Kocher maneuver was performed without partial resection of the prerenal fascia. The further steps of resection did not include removal of the mesojejunum, and the resection of the pancreatic head was achieved in cranio-caudal fashion (not uncinate first). Furthermore, the nerve plexus around the SMA and the periarterial lymphatic vessels were left *in situ* (Inoue level I + II [12]), and the dissection of the SMV was confined to the right hemicircle (180°).

The further steps during surgery, including the reconstruction phase, did not differ between the two groups. Standard reconstruction was performed in all patients, including a two-layer end-to-side pancreatojejunostomy with 5–0 PDS single knots, single-layer end-to-side hepaticojejunostomy with 5–0 PDS single knots, and two-layer end-to-side duodeno- (or gastro-) jejunostomy with 5–0 PDS (or 4–0 PDS) continuous sutures. Postoperative care followed the standard operating procedures and did not differ between the two groups. All patients received standard pancreatic exocrine enzyme substitution therapy from the beginning of oral food intake on according to the standard of care.

Patients that did not undergo surgical tumor resection and those presenting with tumor entities other than PDAC in the frozen section intraoperatively were excluded from the trial and not randomized, as the primary outcome was either not assessable or not comparable with PDAC resection.

### Outcome measures and assessment

The primary endpoint of the trial was the rate of curative resections defined as microscopically complete (>0.1 cm margin clearance; R0) or with a margin of ≤0.1 cm (termed R0(CRM+)) (TNM 8th edition) according to a standardized protocol for pathologic evaluation [8]. The primary endpoint was evaluated postoperatively by a blinded pathologist trained in standardized pathologic specimen handling.

The following secondary endpoints were assessed by physicians of the clinical trials unit:

(1) Rate of superficial and deep surgical site infections (SSI) according to CDC criteria within 30 days after the index operation [18].
(2) Rate of the following pancreatic surgery-associated complications:
– Postoperative pancreatic fistula (POPF) as defined by the ISGPS [19].
– Postpancreatectomy hemorrhage (PPH) as defined by the ISGPS [20].
– Delayed gastric emptying (DGE) as defined by the ISGPS [21].
– Lymphatic fistula/chyle leak as defined by the ISGPS [22].
– Diarrhea as defined and graded by the CTCAE version 4.03 [23].
– Other postoperative complications graded according to the Clavien–Dindo classification [24].
(3) Overall survival within 2 years.
(4) Local recurrence within 2 years.
(5) Quality of life as measured using the EORTC QLQ-C30 and PAN26.
(6) Length of primary hospital stay in days, from day of operation until day of discharge.
(7) Serious adverse events (life-threatening or fatal) in both groups as defined by ICH good clinical practice guidelines E2A (27 October 1994) [25] (12).

### Trial visits

Patients scheduled for elective PD were consecutively screened preoperatively for inclusion and exclusion criteria (visit 1), and patients that had given informed consent were randomized during surgery (visit 2) after the surgeon had confirmed that both interventions could be performed. The specimen was processed via a standardized pathologic protocol by a trained pathologist (visit 3). Patients had planned follow-up visits on postoperative days 5–7, 10–14, and 30–35 (visits 4–6) for evaluation of secondary endpoints. Additionally, at 6, 12, 18, and 24 months after surgery (visits 7–10), follow-up visits were performed to evaluate the long-term results of secondary outcome parameters.

### Statistical analyses

Sample size calculation was based on the primary endpoint [R0 and R0(CRM+) rate on the postoperative specimen] and was conducted using nQuery Advisor^®^ software version 7.0 (Statistical Solutions Ltd, Cork, Ireland). Based on the assumption that in our collective the rate of R0 resections in the control group is 20.0 % [8], 9] and the R0(CRM+) rate is approximately 20.0 % [8], 9], i.e., 40 % combined R0+ R0(CRM+), we hypothesized an increase of 25 % in the experimental intervention arm, as shown in previous studies [12], 13]. Consequently, a group sample size of 82 patients was needed for comparison by the chi-square test, to achieve 90 % power in detecting this difference in R0 + R0(CRM+) rate at a two-sided level of significance of 5 %. Assuming a drop-out rate of up to 10 %, a total of 180 patients (90 per group) were planned to be enrolled in the study.

For the analyses of the outcome measures of the trial, a modified intention to treat set (mITT set) was assembled. The mITT set includes all patients who have completed visit 2 (surgery and randomization), regardless of the treatment they have received. Continuous variables are described in terms of number of missing values, mean, and standard deviation. For binary or categorical variables, absolute and relative frequencies are provided. The confirmatory analysis of the primary endpoint was based on the mITT set. The comparison of rates of curative resections [R0 and R0(CRM+)] between the intervention and control group was conducted with Barnard’s exact test at a 5 % level of significance (two-sided); no imputation of missing values for the primary endpoint in the primary analysis was done. For comparison of the baseline characteristics and the secondary postoperative outcomes between the groups, descriptive two-sided p-values are provided (Wilcoxon U-test for continuous variables and QoL scores, Barnard’s exact test for binary and chi-square test for categorical variables) to show comparability of the groups and to provide a further descriptive measure. P-values <0.05 were defined as statistically significant in a descriptive sense. Analyses were performed using SAS version 9.4.

## Results

### Recruitment and patient distribution

**Figure 1:**
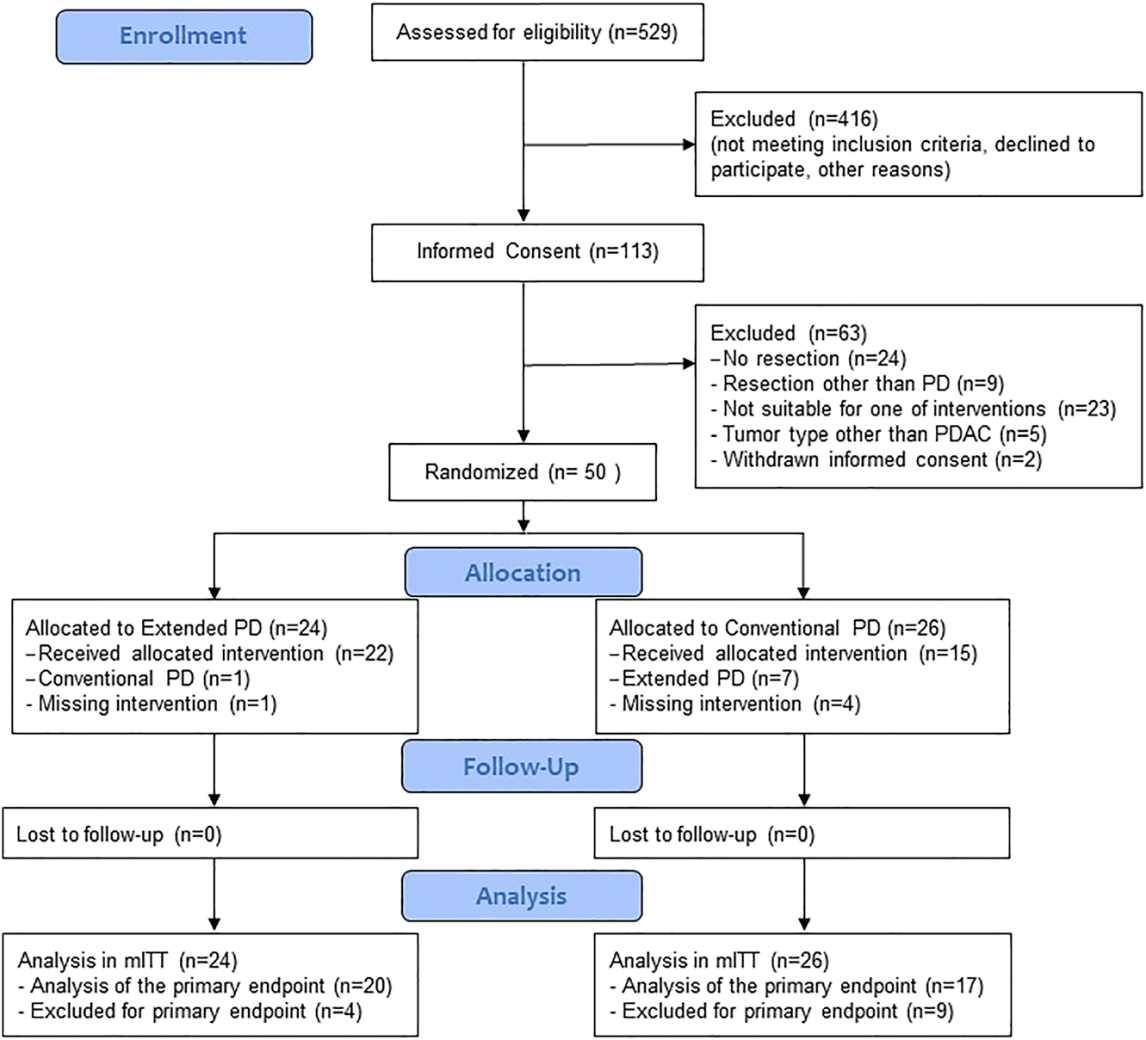
Consort flow chart. PD, pancreatoduodenectomy.

All consecutive patients scheduled for elective PD for suspected PDAC of the pancreatic head at the department of General, Visceral and Transplantation Surgery of Heidelberg University Hospital were screened for eligibility. Between April 4, 2018 and August 19, 2020, a total of 529 patients were screened for eligibility, of whom 113 were included in the trial after informed consent (Figure 1). Patients not included in the trial either did not fulfill the inclusion and exclusion criteria of the trial or declined to participate. Of the included patients, 63 were not randomized intraoperatively as they did not fulfill the intraoperative inclusion criteria. The reasons were as follows: no resection (n=24); resection other than PD, e.g., distal pancreatectomy (n=9); not suitable for one of the interventions, e.g., in the case of vascular infiltration (n=23); tumors other than PDAC in frozen section (n=5); or withdrawal of informed consent before resection (n=2). In line with the study protocol, no further data were recorded and no analyses were performed for these patients. The remaining 50 patients were randomly assigned to either extended PD (n=24) or conventional PD (n=26). Of the 24 patients in the extended PD group, 22 patients received the allocated intervention described above. One patient in the intervention group received the control intervention, and for one patient resection data were missing. In the control group, 15 of the 26 patients received conventional PD. Seven patients in the control group underwent extended resections including Inoue level III, and another four patients had missing data regarding the explicit resection intervention. Tumor types other than PDAC could not be compared regarding the primary endpoint of the trial, the rate of R0 and R0(CRM+) resections. In total, four patients in the extended PD group and nine patients in the conventional PD group exhibited non-PDAC tumors in definitive histology and had to be excluded from the analysis of the primary endpoint but could be included for all other postoperative outcome analyses.

As the recruitment of the PancER trial did not meet expectations, the trial was prematurely terminated on August 19, 2020.

### Patient baseline characteristics

For the analysis of the baseline characteristics, the complete mITT dataset of the randomized patients was analyzed (Table 1). Baseline characteristics were evenly distributed between the two groups, with a few exceptions. There were significantly more men in the conventional PD group (73.1 vs. 45.8 %). Furthermore, more patients in the extended PD group (62.5 %) suffered from preoperative jaundice than in the conventional PD group (26.9 %). Additionally, in the conventional PD group, significantly more patients were active or former smokers. Most of the patients had mild or severe systemic comorbidities as indicated by the ASA classification II (32.7 %) and III (63.3 %). Particularly, 88.2 % of the randomized patients had cardiovascular and 26.5 % had pulmonary preexisting conditions.

**Table 1:** Patient baseline characteristics.

	Extended PD	Conventional PD	Total	p-Value
	n=24	n=26	n=50	

Gender				**0.049**

– Male	11 (45.8 %)	19 (73.1 %)	30 (60.0 %)	
– Female	13 (54.2 %)	7 (26.9 %)	20 (40.0 %)	
Age, years	69.4 ± 10.6	67.9 ± 9.1	68.6 ± 9.8	0.490
Body mass index, kg/m^2^	25.6 ± 4.9	26.0 ± 4.0	25.8 ± 4.4	0.490
ASA status				0.349
– I	0 (0.0 %)	2 (7.7 %)	2 (4.1 %)	
– II	7 (30.4 %)	9 (34.6 %)	16 (32.7 %)	
– III	16 (69.6 %)	15 (57.7 %)	31 (63.3 %)	
– Missing	1	0	1	
Diabetes mellitus	7 (29.2 %)	6 (23.1 %)	13 (26.0 %)	0.624
Preoperative gastric outlet obstruction	4 (16.7 %)	2 (7.7 %)	6 (12.0 %)	0.329
Preoperative jaundice	15 (62.5 %)	7 (26.9 %)	22 (44.0 %)	**0.011**
Preoperative biliary drainage	6 (25.0 %)	8 (30.8 %)	14 (28.0 %)	0.650
Previous abdominal surgery	11 (45.8 %)	15 (57.7 %)	26 (52.0 %)	0.402
Cardiovascular comorbidities	13 (81.3 %)	17 (94.4 %)	30 (88.2 %)	0.233
Pulmonary comorbidities	5 (31.3 %)	4 (22.2 %)	9 (26.5 %)	0.551

Smoking				**0.007**

– Active smoker	1 (4.2 %)	5 (19.2 %)	6 (12.0 %)	
– Former smoker	6 (25.0 %)	14 (53.8 %)	20 (40.0 %)	
– Nonsmoker	17 (70.8 %)	7 (26.9 %)	24 (48.0 %)	

Alcohol consumption				0.956

– Yes, often (daily, almost daily)	3 (12.5 %)	3 (11.5 %)	6 (12.0 %)	
– Yes, occasionally (approximately once a week)	4 (16.7 %)	6 (23.1 %)	10 (20.0 %)	
– Yes, rarely (less than once a week)	12 (50.0 %)	12 (46.2 %)	24 (48.0 %)	
– No	5 (20.8 %)	5 (19.2 %)	10 (20.0 %)	
Preoperative CA19-9, U/mL	495.2 ± 868.9	307.3 ± 463.8	399.3 ± 691.6	0.912

PD, pancreatoduodenectomy. Bold values mean p<0.05.

### Intraoperative measures

The intraoperative parameters showed no differences between the two groups regarding the degree of stomach or venous resection (Table 2). None of the patients required arterial resection as this was an exclusion parameter for the trial. The expertise of the operating surgeons was comparable between the two groups. The Inoue level of arterial dissection performed differed significantly between the groups as it was part of the trial intervention. Two patients in the extended PD group did not receive the extended intervention according to Inoue level III (one patient only level I/II, one patient missing information), whereas 11 patients in the conventional PD group did undergo more radical surgery (n=7) or information about the Inoue level was missing (n=4) (Table 2). No difference in operation time was found between the two groups (360.6 ± 86.2 vs. 384.8 ± 83.0 min; p=0.48). Extended lymph node dissection was not part of the intervention. Consequently, in the extended PD group, a mean of 32.0 ± 12.6 lymph nodes were included in the resection specimen, which was not significantly different from the conventional PD group with a mean of 27.7 ± 9.0 lymph nodes (p=0.32). There was no difference between the groups regarding the TNM status or the grade of differentiation (Supplementary Material 2).

**Table 2:** Intraoperative outcome measures.

	Extended PD	Conventional PD	Total	p-Value
	n=24	n=26	n=50	

Degree of stomach resection				0.352

– Pylorus-preserving	10 (41.7 %)	15 (57.7 %)	25 (50.0 %)	
– Pylorus-resecting	13 (54.2 %)	11 (42.3 %)	24 (48.0 %)	
– Classic PD (1/3 resection of stomach)	1 (4.2 %)	0 (0.0 %)	1 (2.0 %)	
Venous resection performed	6 (25.0 %)	7 (26.9 %)	13 (26.0 %)	0.877
Arterial resection performed	0 (0.0 %)	0 (0.0 %)	0 (0.0 %)	

Degree of contamination of surgery according to CDC classification				0.139

– Clean	3 (12.5 %)	7 (26.9 %)	10 (20.0 %)	
– Clean-contaminated	21 (87.5 %)	17 (65.4 %)	38 (76.0 %)	
– Contaminated	0 (0.0 %)	2 (7.7 %)	2 (4.0 %)	

Experience of the surgeon performing the resection				0.729

– <50 Whipple procedures	9 (37.5 %)	11 (42.3 %)	20 (40.0 %)	
– ≥50 Whipple procedures	15 (62.5 %)	15 (57.7 %)	30 (60.0 %)	

Inoue level of arterial dissection performed				**<0.001**

– Level 1/2	1 (4.3 %)	15 (68.2 %)	16 (35.6 %)	
– Level 3	22 (95.7 %)	7 (31.8 %)	29 (64.4 %)	
– Missing	1	4	5	
Duration of operation (min.)	360.6 ± 86.2	384.8 ± 83.0	373.2 ± 84.6	0.478
Number of retrieved lymph nodes	32.0 ± 12.6	27.7 ± 9.0	29.7 ± 10.9	0.321

PD, pancreatoduodenectomy. Bold values mean p<0.05.

### Primary endpoint

For the primary endpoint, patients with tumors other than PDAC had to be excluded, as the R status and CRM are comparable for PDAC resections only and not for other malignant (e.g., neuroendocrine tumors) or benign lesions. Therefore, after exclusion of these patients for the evaluation of the primary endpoint, 20 patients of the extended PD group were included in the analysis, of which 75.0 % were R0 or R0(CRM+) resected in definitive histology. In the conventional PD group, 64.7 % of the 17 patients included had R0 or R0(CRM+) status (Table 3). This difference was not statistically significant (p=0.59).

**Table 3:** Analysis of the primary endpoint.

	Extended PD	Conventional PD	Total	p-Value
	n=20	n=17	n=37	

Primary endpoint (R status)				

– R0/R0(CRM+)	15 (75.0 %)	11 (64.7 %)	26 (70.3 %)	0.587
– R1/R2	5 (25.0 %)	6 (35.3 %)	11 (29.7 %)	

PD, pancreatoduodenectomy; CRM, circumferential resection margin.

### Secondary outcome measures

The analyses of the secondary outcomes were performed with the complete mITT dataset (Table 4). There were no differences between the two groups regarding length of hospital stay, surgical site infections, delayed gastric emptying, or lymphatic fistula (Table 4). In the extended PD group, two POPF grade B occurred and one patient had PPH, whereas in the conventional PD group, two POPF grade B, one POPF grade C, and one PPH were found. Analysis of all pancreas-specific complications showed no significant differences between the two groups (Table 4). In addition, all other postoperative complications as assessed via the Clavien–Dindo classification were evenly distributed between the two groups (Table 4) However, patients in the extended PD group showed a trend toward slightly higher rates of diarrhea on clinical examination, with 29.2 % grade 1, 20.8 % grade 2, and no grade 3 diarrhea vs. 23.1 % grade 1, no grade 2, and 3.8 % grade 3 diarrhea in the conventional PD group (p=0.06).

**Table 4:** Secondary outcome measures.

	Extended PD	Conventional PD	Total	p-Value
	n=24	n=26	n=50	

Length of primary hospital stay, days	20.1 ± 19.3	16.0 ± 9.1	18.0 ± 14.9	0.471

Surgical site infection (SSI)				0.204

– Superficial incisional SSI	1 (4.2 %)	5 (19.2 %)	6 (12.0 %)	
– Deep incisional SSI	1 (4.2 %)	0 (0.0 %)	1 (2.0 %)	

Postoperative pancreatic fistula (POPF)				0.371

– Grade B	2 (8.3 %)	2 (7.7 %)	4 (8.0 %)	
– Grade C	0 (0.0 %)	1 (3.8 %)	1 (2.0 %)	
Postpancreatectomy hemorrhage, PPH	1 (4.2 %)	1 (3.8 %)	2 (4.0 %)	0.954

Delayed gastric emptying, DGE				0.666

– Grade A	2 (8.3 %)	4 (15.4 %)	6 (12.0 %)	
– Grade B	3 (12.5 %)	2 (7.7 %)	5 (10.0 %)	
– Grade C	0 (0.0 %)	0 (0.0 %)	0 (0.0 %)	

Lymphatic fistula/chyle leak				0.992

– Grade A	3 (12.5 %)	3 (11.5 %)	6 (12.0 %)	
– Grade B	1 (4.2 %)	1 (3.8 %)	2 (4.0 %)	

Diarrhea				0.055

– Grade 1	7 (29.2 %)	6 (23.1 %)	13 (26.0 %)	
– Grade 2	5 (20.8 %)	0 (0.0 %)	5 (10.0 %)	
– Grade 3	0 (0.0 %)	1 (3.8 %)	1 (2.0 %)	

Other complications				0.749

– No	10 (41.7 %)	12 (46.2 %)	22 (44.0 %)	
– Yes	14 (58.3 %)	14 (53.8 %)	28 (56.0 %)	

PD, pancreatoduodenectomy.

In the quality of life assessment (EORTC QLQ-C30 and PAN 26), patients treated with extended PD reported significantly more problems with diarrhea on postoperative day 30–35 than those in the conventional PD group (p<0.01) (Table 5). Additionally, when comparing the difference between postoperative day 30–35 to baseline, patients in the extended PD group exhibited significantly reduced physical functioning (p<0.01) and higher rates of appetite loss (p=0.03), nausea/vomiting (p=0.03), and altered bowel habit (p=0.03) than patients with conventional PD. No other aspects of the EORTC QLQ-C30 and PAN 26 differed between the two groups (Table 5).

**Table 5:** Quality of life assessment.

	At postoperative day 30–35				Difference to baseline			
	Extended PD	Conventional PD	Total	p-Value	Extended PD	Conventional PD	Total	p-Value
	n=24	n=26	n=50		n=24	n=26	n=50	

**EORTC QLQ-C30**								

Physical functioning	41.5 ± 22.0	49.1 ± 22.5	45.3 ± 22.3	0.387	−40.7 ± 31.7	−16.9 ± 29.2	−28.4 ± 32.3	**0.007**
Role functioning	32.5 ± 28.6	32.5 ± 21.1	32.5 ± 24.8	0.869	−33.3 ± 40.8	−27.5 ± 29.4	−30.3 ± 35.0	0.757
Emotional functioning	50.0 ± 25.6	52.8 ± 24.9	51.4 ± 25.0	0.842	−13.0 ± 19.0	0.0 ± 22.4	−6.5 ± 21.5	0.199
Cognitive functioning	72.8 ± 23.7	66.7 ± 24.9	69.8 ± 24.2	0.428	−10.4 ± 16.0	−8.3 ± 18.3	−9.4 ± 16.9	0.693
Social functioning	32.5 ± 29.1	32.4 ± 29.4	32.4 ± 28.9	1.000	−31.3 ± 29.7	−25.0 ± 40.4	−28.1 ± 35.0	0.596
Global health status	42.5 ± 23.4	46.8 ± 16.5	44.6 ± 20.1	0.601	−14.4 ± 19.0	−8.3 ± 25.3	−11.3 ± 22.3	0.298
Fatigue	71.3 ± 22.3	67.0 ± 19.6	69.2 ± 20.9	0.487	29.2 ± 32.7	18.6 ± 24.1	23.7 ± 28.6	0.170
Nausea/vomiting	21.9 ± 24.9	14.9 ± 20.7	18.4 ± 22.9	0.279	18.8 ± 24.2	3.9 ± 17.2	11.1 ± 21.9	**0.033**
Pain	30.7 ± 25.0	41.2 ± 27.4	36.0 ± 26.4	0.163	3.1 ± 32.9	8.8 ± 40.0	6.1 ± 36.3	1.000
Dyspnea	45.6 ± 35.5	33.3 ± 33.3	39.5 ± 34.5	0.274	13.3 ± 41.4	−5.9 ± 42.9	3.1 ± 42.6	0.220
Insomnia	45.6 ± 25.4	40.4 ± 26.2	43.0 ± 25.6	0.648	14.6 ± 38.4	5.9 ± 37.7	10.1 ± 37.7	0.653
Appetite loss	73.7 ± 30.6	56.1 ± 29.5	64.9 ± 30.9	0.065	50.0 ± 36.5	17.6 ± 42.7	33.3 ± 42.5	**0.032**
Constipation	1.8 ± 7.6	17.5 ± 34.0	9.6 ± 25.6	0.073	−8.3 ± 22.8	0.0 ± 31.2	−4.0 ± 27.3	0.412
Diarrhea	71.9 ± 31.9	33.3 ± 39.6	53.2 ± 40.4	**0.005**	54.2 ± 46.9	−4.4 ± 56.2	25.8 ± 58.8	**0.010**
Financial problems	22.8 ± 29.5	25.9 ± 33.4	24.3 ± 31.1	0.893	15.6 ± 30.5	6.3 ± 27.8	10.8 ± 29.0	0.331

**EORTC PAN26**								

Pancreatic pain	36.0 ± 22.7	42.1 ± 17.5	39.0 ± 20.2	0.353	3.3 ± 26.0	4.7 ± 32.5	4.0 ± 29.0	0.955
Digestive symptoms	74.6 ± 29.6	65.8 ± 24.5	70.2 ± 27.2	0.212	34.4 ± 30.1	16.7 ± 42.2	25.5 ± 37.1	0.122
Altered bowel habit	65.8 ± 29.6	54.4 ± 36.4	60.1 ± 33.2	0.342	37.5 ± 30.1	3.9 ± 44.7	20.2 ± 41.4	**0.033**
Hepatic	18.4 ± 26.6	9.6 ± 17.0	14.0 ± 22.4	0.288	−13.5 ± 30.6	−19.8 ± 28.7	−16.7 ± 29.3	0.939
Body image	51.8 ± 31.4	51.8 ± 28.3	51.8 ± 29.5	0.976	28.1 ± 34.8	12.7 ± 26.7	20.2 ± 31.4	0.175
Satisfaction with health care	75.0 ± 20.8	79.8 ± 28.6	77.5 ± 24.9	0.267	−2.2 ± 42.2	−7.8 ± 28.9	−5.2 ± 35.3	0.663
Sexuality	39.3 ± 46.0	50.9 ± 39.8	45.8 ± 42.3	0.477	−33.3 ± 43.0	−6.7 ± 40.7	−17.3 ± 42.9	0.138

PD, pancreatoduodenectomy. Bold values mean p<0.05.

### Long-term follow-up

The long-term follow-up of patients performed at 6, 12, 18, and 24 months after surgery revealed no differences in the overall survival or the rates of local recurrence between the two groups. Overall survival was 58.3 % in the extended PD group and 57.7 % in the conventional PD group 2 years after surgery (p=0.90). Five patients in the extended PD group and six patients in the conventional PD group suffered from local recurrence 2 years after surgery (p=0.69). In the quality of life assessment, no significant differences regarding diarrhea, nausea/vomiting, bowel habit, and appetite loss could be found between the two groups at any of the long-term follow-up visits.

## Discussion

The main objective of the PancER trial was to investigate the effect on the rate of R0 resections of extended pancreatic head resection for the treatment of PDAC vs. conventional PD. Furthermore, data on perioperative outcomes were collected to elucidate the feasibility and safety of the PancER procedure. In the PancER trial, extended PD consisted of a combination of a modified Kocher maneuver as described by Weitz et al. with partial resection of the prerenal fascia [17], an uncinate first approach as described by Hackert et al. [13], and SMD along the SMA (≥180°) equivalent to level III dissection according to Inoue et al. [12]. The results of the trial show that the PancER procedure is feasible and can be performed safely with comparable perioperative outcomes. Patients in both groups had comparable postoperative complications according to Clavien–Dindo. However, patients self-reported significantly more problems with diarrhea in the extended PD group at short-term follow-up. This difference between the two groups disappeared in the long-term follow-up. On the other hand, in the extended PD group, R0 resections were 10 % more frequent than in the conventional PD group (75.0 vs. 64.7 %), which was not statistically significant.

Previous trials investigating extended PD for PDAC focused mainly on the extent of lymph node dissection. These trials and subsequent meta-analyses showed that extended lymphadenectomy during PD does not improve overall survival but leads to higher rates of morbidity [26], [27], [28]. In contrast to these trials, the extended PD intervention investigated in the PancER trial did not primarily aim to enhance lymphadenectomy but focused on the extent of resection along the soft tissue margins of the SMA and the retroperitoneum, where, owing to the perineural growth pattern of PDAC, R1 resections are most frequent [8], 10]. Only two other randomized trials could be identified that investigated extended PD similar to the PancER trial intervention. Jang et al. investigated the effect of extended PD compared with conventional PD and found no significant survival benefit but comparable perioperative outcomes [29]. The intervention of this trial included extended lymphadenectomy and “celiac and SMA nerve plexus dissection” on the “right side.” The extent of the latter remained unclear. The other trial describing an intervention similar to the PancER trial is the Maple-PD trial [30]. A study protocol for this trial was published in 2018, but the results are still pending. Thus, the PancER trial is one of the first randomized trials to investigate radical dissection along the SMA during PD.

Regarding the effect of extended PD on the R0 resection rate, the PancER trial could not find a significant difference between the two groups with an absolute difference of somewhat more than 10 % in favor of the extended PD group. This difference was below the 25 % difference that we assumed for sample size calculation. Several factors may have combined to account for this. First, in contrast to the planned randomization of 90 patients per group, only 50 patients were randomized and only 37 patients were available for the analysis of the primary endpoint. This was due to the strict inclusion and exclusion criteria of the study, e.g., excluding all patients with prior neoadjuvant treatment. Consequently, more than 500 patients were screened to achieve randomization of 50 patients (ratio 1:10). Future studies should address this problem by using more liberal inclusion/exclusion criteria. Second, in this single-center study, patients in the control group exhibited higher rates of R0 or R0(CRM+) resections (64.7 %) than was assumed for the control group based on previous reports [12], 13]. This reflects on the one hand the expertise of the high-volume center in Heidelberg and on the other hand the fact that a subgroup of patients in the control group underwent radical resection along the SMA despite being randomized into the control group (Table 2).

The increased rate of diarrhea reported by patients was in line with an, albeit not significant, increase in the rate of diarrhea on clinical examination. These results are in accordance with previous reports investigating extended resections along the SMA for PDAC, which showed an increase in intractable diarrhea [31]. In the PancER trial, all patients received standard pancreatic exocrine enzyme substitution therapy from the beginning of oral food intake on according to the standard of care. Therefore, in line with the literature, the diarrhea is caused by removing part of the nerve plexus around the SMA in level III SMD. The long-term effects of this symptom are unclear, as our current study only addresses short-term outcomes and did not compare the frequency of adjuvant treatment between the two study arms, which might be affected by severe diarrhea. However, recent studies have shown that starting adjuvant chemotherapy in PDAC late is just as effective as early initiation of chemotherapy [32], and current guidelines recommend beginning adjuvant chemotherapy at any time in the first 12 weeks after surgery [16]. Therefore, patients undergoing more radical resection have enough time to recover fully before initiation of adjuvant chemotherapy. In the long-term follow-up of the PancER trial, the difference between the two groups regarding the rate of diarrhea disappeared starting from 6 months after surgery.

There are several limitations of the trial. First, the rate of complete resections (primary endpoint) only acts as a surrogate parameter for survival. However, several reports have established R1 resection as a major predictor for survival. In addition, in the PancER trial, a relevant number of patients with LAPC and BRPC were included preoperatively, resulting in a high rate of patients in whom intraoperative assessment showed that conventional PD was not suitable and in fact extended PD was indicated. Furthermore, biopsy-proven PDAC was not a prerequisite for preoperative inclusion into the trial, leading to a relevant number of patients being found to have tumors other than PDAC on final histology. For these patients, CRM assessment was not applicable. Therefore, tumor entities similar to PDAC, such as intraductal papillary mucinous neoplasia (IPMN)-associated carcinoma and periampullary carcinoma should be considered for inclusion in future trials.

In order to enhance the extent of surgical tumor resection further, an even more radical intervention termed the “TRIANGLE” operation has been developed at Heidelberg University Hospital [33], 34]. The TRIANGLE procedure involves not only radical resection along the SMA but also removal of all lymphatic, nerve, and soft tissue in the “TRIANGLE” region between the SMA, the CA, and the mesenterico-portal axis. As recruitment of the PancER trial failed to meet expectations and the retrospective analysis of the TRIANGLE intervention showed a promising difference in the rate of complete resections [34], recruitment of the PancER trial was prematurely terminated on August 19, 2020. The results of the PancER trial served as data for a current large multicenter trial investigating survival after the TRIANGLE operation in comparison with conventional PD [35].

In summary, the PancER trial showed that extended PD can be performed safely in a randomized-controlled trial setting at a high-volume center, with comparable immediate perioperative outcomes.

## Conclusions

The PancER trial shows that extended PD with more radical resection along the SMA can be performed with perioperative outcomes comparable with those of conventional PD. Although the intervention improved the R0 resection rate by 10 %, this increase was below expectations. Therefore, an even more radical PD resection technique involving not only the SMA but also the celiac and hepatic artery (TRIANGLE operation) was developed at Heidelberg University. The TRIANGLE operation is currently being evaluated in a randomized controlled multicenter trial [35]. The results of the PancER trial served as pilot data for this subsequent study.

## Supplementary Material

Supplementary Material
