# Supplementary material for: Conventional partial pancreatoduodenectomy versus an uncinate first, extended partial pancreatoduodenectomy approach for the resection of pancreatic head cancer: the randomized, controlled PancER trial
Source: Innov Surg Sci. 2024 Aug 26;10(3):109–19. doi: 10.1515/iss-2024-0014 (PMC12552034; doi:10.1515/iss-2024-0014)
Supplement: Supplementary file 1 — Supplementary Material [file j_iss-2024-0014_suppl_001.pdf]

Conventional partial pancreatoduodenectomy *versus* an uncinate first, extended partial pancreatoduodenectomy approach for the resection of pancreatic head cancer: the randomized, controlled PancER trial

Patrick Heger, Markus K. Diener, Manuel Feißt, Matthias M. Gaida, Christina Klose, Phillip Knebel, Rosa Klotz, Colette Dörr-Harim and André L. Mihaljevic

Supplementary material

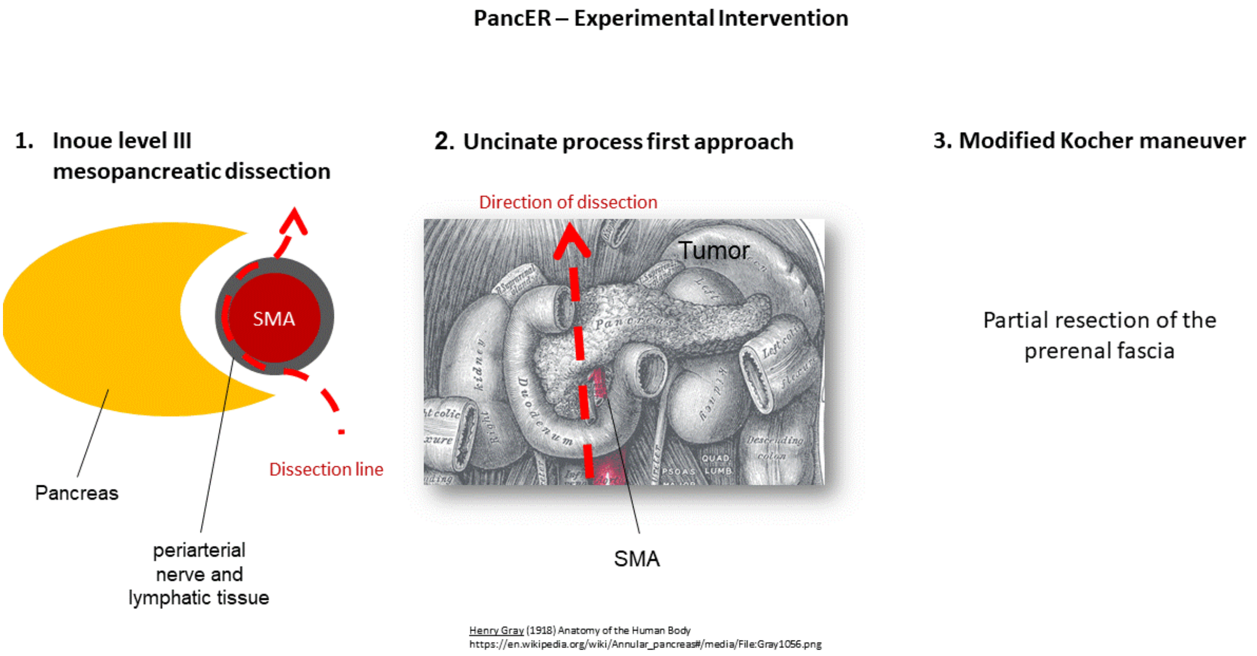

Supplement 1.1

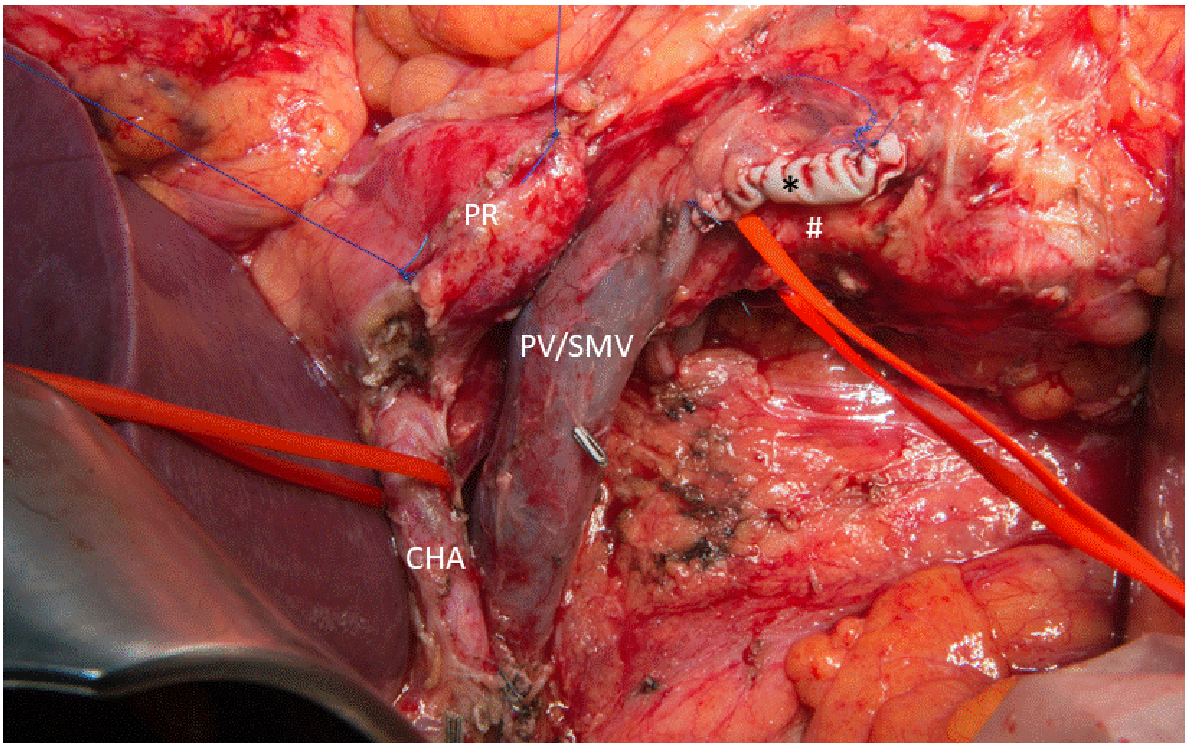

* peritoneal patch on SMV
# superior mesenteric artery

CHA: common hepatic artery
PR: pancreatic remnant

Supplement 1.2

	Extended PD	Conventional PD	Total	p-value
	N=24	N=26	N=50	
TNM classification (TNM; 8th edition)				
pT classification				0.364
- pT1	2 (9.%)	2 (8.3%)	4 (8.7%)	
- pT2	11 (50.0%)	16 (66.7%)	27 (58.7%)	
- pT3	9 (40.9%)	5 (20.8%)	14 (30.4%)	
- pT4	0 (0.0%)	1 (4.2%)	1 (2.2%)	
- missing	2	2	4	
pN classification				0.690
- pN0	4 (18.2%)	7 (28.0%)	11 (23.4%)	
- pN1	9 (40.9%)	8 (32.0%)	17 (36.2%)	
- pN2	9 (40.9%)	10 (40.0%)	19 (40.4%)	
- missing	2	1	3	
pM classification				0.900
- pM1	1 (4.8%)	1 (4.0%)	2 (4.3%)	
- no statement possible (cM0)	20 (95.2%)	24 (96.0%)	44 (91.1%)	
- missing	3	2	5	
Perineural tumor propagation				0.363
- Pn0	1 (4.8%)	3 (12.5%)	4 (8.9%)	
- Pn1	20 (95.2%)	21 (87.5%)	41 (91.1%)	
- missing	3	2	5	
Vascular invasion				0.163
- V0	7 (31.8%)	13 (52.0%)	20 (42.6%)	
- V1	15 (68.2%)	12 (48.0%)	27 (57.4%)	
- missing	2	1	3	
Lymphatic tumor propagation				0.368
- L0	3 (13.6%)	6 (24.0%)	9 (19.1%)	
- L1	19 (86.4%)	19 (76.0%)	38 (80.9%)	
- missing	2	1	3	
Grade of differentiation according to WHO				0.356
- G1	0 (0.0%)	1 (4.0%)	1 (2.1%)	
- G2	14 (63.6%)	16 (64.0%)	30 (63.8%)	
- G3	8 (36.4%)	6 (24.0%)	14 (29.8%)	
- G4	0 (0.0%)	2 (8.0%)	2 (4.3%)	
- missing	2	1	3	

Supplement 2
